# Takotsubo Cardiomyopathy: An Exploration of the Intersection Between Stress, Coronary Dysfunction, and Cardiac Outcomes

**DOI:** 10.31083/RCM45857

**Published:** 2025-12-23

**Authors:** Davide Rossi, Silvio Saraullo, Roberta Magnano, Laura Pezzi, Alberto D'Alleva, Fabrizio Ricci, Claudio Scollo, Mario Di Marino, Eugenio Genovesi, Piergiusto Vitulli, Daniele Forlani, Giulia Renda, Sabina Gallina, Massimo Di Marco

**Affiliations:** ^1^Department of Neuroscience, Imaging and Clinical Sciences, G. D'Annunzio University of Chieti-Pescara, 66100 Chieti, Italy; ^2^University Cardiology Division, Heart Department, “SS Annunziata” Polyclinic University Hospital, 66100 Chieti, Italy; ^3^Cardiology and ICCU Department, Santo Spirito Hospital, 65124 Pescara, Italy; ^4^Department of Clinical Sciences, Lund University, 21428 Malmö, Sweden

**Keywords:** Takotsubo syndrome, stress cardiomyopathy, coronary artery disease, microvascular dysfunction, neurohormonal dysregulation, cardiovascular imaging

## Abstract

Takotsubo syndrome (TTS) is an acute, reversible form of left ventricular dysfunction, typically triggered by emotional or physical stress. The hallmark feature is reversible regional wall motion abnormality extending beyond a single coronary distribution, most commonly presenting with an apical ballooning pattern. The pathophysiology is multifactorial, encompassing neurohormonal dysregulation, catecholamine-mediated toxicity, microvascular dysfunction, oxidative stress, inflammation, and metabolic disturbances. Nonetheless, despite growing recognition, an evidence gap persists in diagnosing TTS. Meanwhile, TTS is classified within myocardial infarction with non-obstructive coronary arteries (MINOCAs) and frequently treated as a diagnosis of exclusion. Further complicating the diagnostic algorithm, emerging evidence indicates that TTS and coronary artery disease (CAD) may coexist, suggesting a potential bidirectional relationship rather than a bystander phenomenon. Moreover, TTS shares several pathophysiological mechanisms with coronary microvascular dysfunction syndromes: angina with non-obstructive coronary arteries (ANOCAs) and ischemia with non-obstructive coronary arteries (INOCAs). These overlaps underscore the need for rigorous differential diagnosis and careful comprehensive evaluation of hemodynamic significance, plaque morphology, and microvascular phenotyping to enhance clinical recognition and optimize therapeutic decision-making. This review synthesizes current evidence on the diagnosis and management of TTS, emphasizing the intersection between TTS and coronary and microvascular disorders to promote a more targeted, mechanism-based therapeutic approach.

## 1. Introduction

Takotsubo syndrome (TTS) is an acute cardiac condition characterized by transient left ventricular (LV) dysfunction, typically occurring in the absence of significant obstructive coronary artery disease (CAD). TTS commonly affects postmenopausal women and is frequently triggered by emotional or physical stress. Clinically, TTS mimics acute coronary syndrome (ACS) but shows no obstructive coronary lesions on angiography with regional wall motion abnormality extending beyond a single coronary artery territory. The underlying pathophysiology is poorly understood and involves a neurocardiogenic interplay [1, 2]. Although generally reversible, TTS is associated with significant short- and long-term complications, including arrhythmias, heart failure (HF), and a recurrence rate of approximately 20% over ten years [3, 4].

**Fig. 1.**
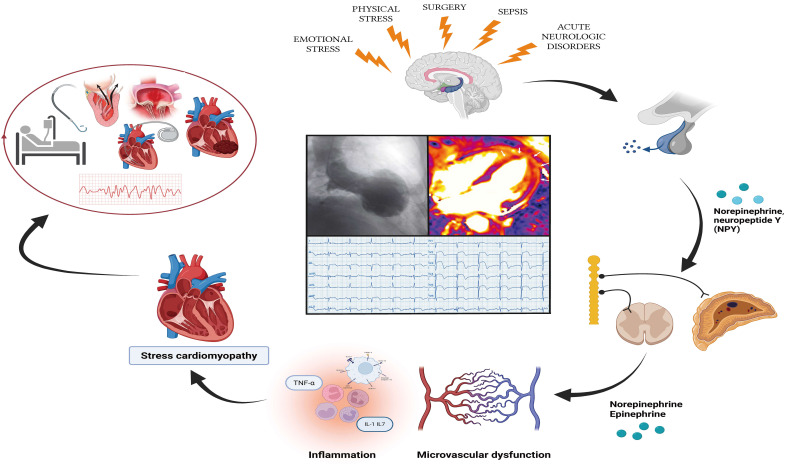
**Pathophysiological relationship between risk factors, TTS, and clinical outcomes (central illustration)**. IL-1, interleukin 1; IL-17, interleukin 17; NPY, neuropeptide Y; TNF-α, tumor necrosis factor alpha. Figure created with BioRender.com.

Recent advances, such as the InterTAK diagnostic criteria and myocardial tissue characterization on cardiac magnetic resonance (CMR), have improved diagnostic accuracy [5]. However, major challenges persist, particularly in differentiating TTS from the spectrum of myocardial infarction with non-obstructive coronary arteries (MINOCAs), in patients with concomitant CAD, or in those with microvascular dysfunction (angina with non-obstructive coronary arteries (ANOCAs)/ischemia with non-obstructive coronary arteries (INOCAs)). Current evidence on the interplay between TTS and coronary disorders remains limited, although data suggest a bidirectional relationship likely mediated by microvascular dysfunction. Nevertheless, therapeutic strategies are still largely empirical and require further validation (Fig. 1).

This review conducted a comprehensive literature review using the PubMed, Scopus, and Web of Science databases, focusing on studies addressing stress cardiomyopathy and TTS.

## 2. Definition

TTS is characterized by an acute yet reversible LV systolic dysfunction, typically resolving within three weeks. The onset of TTS often follows an emotional or physical stressor within the preceding few days. TTS is defined by regional LV wall motion abnormalities that extend beyond a single coronary artery territory, most commonly apical akinesia, in the absence of obstructive CAD [2].

The most widely adopted criteria for TTS (Table 1, Refs. [4, 5, 6]) are those proposed by the Heart Failure Association of the European Society of Cardiology for Takotsubo Syndrome [6], which refines the earlier Mayo Clinic Criteria [7]. More recently, the International Takotsubo Diagnostic Criteria (InterTAK Diagnostic Criteria) have been introduced, incorporating clinical variables such as triggers, electrocardiogram (ECG) findings, and patient demographics to improve diagnostic specificity and sensitivity [5, 8].

**Table 1.** **Diagnostic criteria for TTS according to ESC, InterTAK, and Mayo Clinic criteria [4, 5, 6]**.

International Takotsubo Diagnostic Criteria (InterTAK)	
	- Transient left ventricular systolic dysfunction is a hallmark, typically characterized by hypokinesia, akinesia, or dyskinesia and presenting as apical ballooning or midventricular, basal, or focal wall motion abnormalities. In some cases, the right ventricle may also be affected.
	- Emotional, physical, or combined stressors often precede the onset of TTS, although the occurrence of such triggers is not obligatory.
	- Neurological disorders, including subarachnoid hemorrhage, stroke/transient ischemic attack, or seizures, and pheochromocytoma, can also precipitate TTS episodes.
	- New electrocardiographic alterations such as ST-segment elevation, ST-segment depression, T-wave inversion, or corrected QT interval (QTc) prolongation, although exceptional cases without notable ECG changes have been reported.
	- Moderately elevated cardiac biomarkers.
	- The presence of significant coronary artery disease does not preclude a diagnosis of TTS.
	- There is no clinical or laboratory-based evidence indicative of infectious myocarditis in these patients.
European Society of Cardiology Criteria	
	- Transient, region-specific dysfunction affecting the left or right ventricular wall, frequently—but not invariably—triggered by an emotional or physical stressor.
	- Wall motion abnormalities that typically span more than one epicardial vascular territory, often culminating in circumferential dysfunction of the involved ventricular segments.
	- Exclusion of significant atherosclerotic coronary artery lesions, such as acute plaque rupture, thrombosis, or dissection, or any alternative structural or pathological process (e.g., hypertrophic cardiomyopathy, viral myocarditis), which could otherwise account for the observed, temporary ventricular dysfunction.
	- Novel, reversible ECG alterations, including ST-segment elevation, ST-segment depression, left bundle branch block, T-wave inversion, and/or QTc prolongation, arising during the acute phase (up to three months).
	- Markedly elevated serum natriuretic peptides (B-type natriuretic peptide (BNP) or N-terminal pro B-type natriuretic peptide (NT-proBNP)) in the acute setting.
	- Elevated cardiac troponin levels measured using conventional assays, although generally modest and out of proportion to the extent of observed myocardial dysfunction.
	- Restoration of normal systolic function on follow-up cardiac imaging (within three to six months).
Revised Mayo Clinic Criteria	
	- Temporary hypokinesis, akinesis, or dyskinesis primarily involving the midventricular segments of the left ventricle, with or without apical involvement; in most cases, these regional wall motion abnormalities extend beyond a single epicardial vascular territory. A stress-related trigger is frequently, but not universally, implicated.
	- No evidence of obstructive coronary artery disease or angiographic signs pointing to acute plaque rupture.
	- New electrocardiographic changes (e.g., ST-segment elevation and/or T-wave inversion) or mild elevations in cardiac troponin levels.
	- Exclusion of pheochromocytoma and myocarditis as potential causes.

## 3. Epidemiology and Risk Factors 

The estimated prevalence of TTS ranges from 15 to 30 cases per 100,000 individuals annually; however, the true incidence is likely higher, as TTS cases are misclassified as ACS, accounting for 1–2% of ACS presentations [1, 2, 5, 9].

In the German Italian Spanish Takotsubo (GEIST) registry, which included 2492 patients, nearly 90% were women, predominantly perimenopausal (mean age: 69 ± 13 years) and with higher comorbidities [10]. The susceptibility of this patient group appears to be related to heightened sympathetic overactivation, endothelial dysfunction (estrogen-related), and increased neuropeptide Y levels following menopause. Emotional stress is a more frequent trigger in women, whereas men are more often affected by physical stressors and exhibit higher risk phenotypes [11, 12].

Pediatric TTS remains rare (3.1 per 100,000 discharges), predominantly affecting males, who display lower ejection fraction and higher rates of cardiogenic shock than adults, although outcomes are comparable [13, 14].

TTS has also been reported during cancer therapy, particularly carboplatin [15], paclitaxel, 5-fluorouracil (5-FU), and immune checkpoint inhibitors [16]. In a meta-analysis of 41 case reports, 5-FU accounted for over one-third [17], likely through direct cardiotoxicity mediated by free radical-induced myocyte injury [15, 18].

Additionally, TTS may occur as part of the stroke–heart syndrome (SHS) following acute neurological events such as seizures, ischemic stroke, or intracranial hemorrhage, reflecting shared autonomic network dysfunction [19, 20].

In a cohort of 2300 TTS patients, 17% had a concomitant neurological disorder, which was associated with longer hospital stays and higher complication rates [21]. Although most cases are sporadic, familial clustering has been described, suggesting a potential genetic predisposition; adrenergic receptor polymorphisms and estrogen receptor variants may enhance susceptibility to catecholamine surges [22].

## 4. Pathophysiology and Mechanisms of Stress Cardiomyopathy

TTS represents a unique model of neurocardiogenic injury: emotional or physical stress precipitates transient myocardial dysfunction (Fig. 1) [23, 24].

The exact pathophysiology of TTS remains uncertain and appears multifactorial. Emotional or physical stressors, including acute illness, surgery, pain, sepsis, substance abuse, and exacerbations of chronic obstructive pulmonary disease (COPD), are common triggers; neurological conditions such as seizures, stroke, or head trauma are also implicated [25]. In some cases, positive emotional events—happy heart syndrome—can precipitate TTS. However, no clear trigger was identified in up to one-third of cases, suggesting a role for psychosocial vulnerability such as coping mechanisms and social support [26].

Neuroimaging studies reveal transient limbic dysfunction and altered cerebral blood flow in the hippocampus and basal ganglia during acute TTS, which normalize upon recovery [27, 28, 29]. These findings support the concept of a brain–heart axis, in which stress activates the limbic system, locus coeruleus, and hypothalamic–pituitary–adrenal (HPA) axis. The resulting release of norepinephrine and neuropeptide Y induces coronary microvascular and epicardial dysregulation, particularly in individuals with CAD, owing to an inappropriate response to a catecholamine surge [6, 23, 24, 30]. Estrogen deficiency amplifies sympathetic tone and attenuates endothelial nitric oxide signaling, explaining the female predominance [23].

**Fig. 2.**
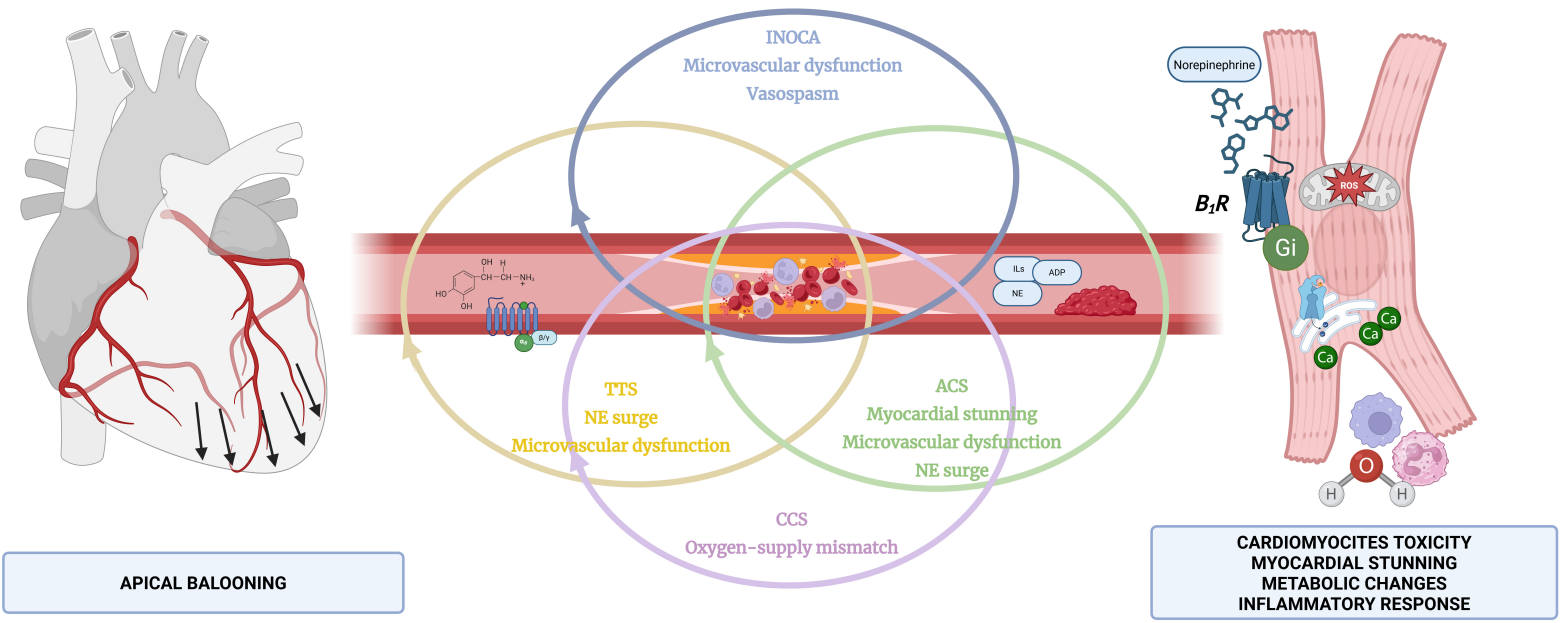
**Interdependence between TTS, ACS, chronic coronary syndrome (CCS) with CAD, and microvascular dysfunction disorders, such as INOCA**. ILs, interleukins; ADP, adenosine diphosphate; NE, norepinephrine; Ca, calcium; ROS, reactive oxygen species; B_1_R, beta-adrenergic receptor. Figure created with BioRender.com.

Excessive β-adrenergic receptor (βAR) stimulation induces intracellular calcium overload, oxidative stress, and mitochondrial dysfunction, leading to transient myocardial stunning (Fig. 2). Indeed, the densities of β_1_AR and β_2_AR are highest at the apex. In contrast, sympathetic innervation is strongest in the basal LV and weakest in the apex, making the apical myocardium more vulnerable to catecholamine toxicity. At high catecholamine concentrations, β_2_AR shifts from a stimulatory Gs to an inhibitory Gi signaling pathway, producing negative inotropy and apical akinesia [23]. Evidence from metaiodobenzylguanidine (MIBG) imaging also shows regional sympathetic denervation during the acute phase [23, 24].

Evidence supports a role for coronary microvascular dysfunction (CMD) and endothelial dysregulation. TTS patients exhibit impaired perfusion, vasospasm, and abnormal release of vasoactive mediators, including endothelin-1, von Willebrand factor, and plasminogen activators [24, 25, 30, 31].

At the myocardial level, catecholamine-mediated toxicity triggers oxidative stress, mitochondrial injury, and inflammation. Inflammatory cell infiltration, with elevated C-reactive protein (CRP) and interleukin-6 (IL-6), contributes to myocardial edema and microvascular impairment. Metabolically, decreased free fatty acid utilization and glucose uptake in apical segments indicate a shutdown of mitochondrial metabolism [6, 24, 25, 30, 32].

## 5. Clinical Classification

Primary TTS refers to cases in which the condition leads patients to seek medical attention, whereas secondary TTS occurs in individuals already hospitalized for another acute illness [24]. However, the distinction is often blurred, as overlapping clinical presentations can complicate the diagnosis.

Anatomically, TTS can be classified into three main types (Table 2): apical (75–80% of cases), midventricular (10–20%), and basal (<5%). The midventricular form is typically associated with more severe reductions in cardiac output and a higher incidence of cardiogenic shock. Rare forms, including biventricular dysfunction or isolated right ventricular involvement, are also described and often present with hemodynamic instability [2, 24]. However, the mechanism underlying these anatomical differences remains incompletely understood, although regional autonomic dysfunction likely plays a pivotal role.

**Table 2.** **Anatomical classifications of TTS**.

Type	Prevalence (%)	Characteristics
Apical	75–80	Most frequent type.
Midventricular	10–20	Hypokinesia or akinesia in the mid-left ventricle; normal contraction in apical and basal regions.
Basal (inverted)	<5	Associated with less severe hemodynamic disturbances.
Biventricular	Uncommon	Linked to significant hemodynamic instability and shock.
Isolated right ventricular involvement	Uncommon	Linked to significant hemodynamic instability and shock.
Localized focal dysfunction	Uncommon	More benign.

## 6. Diagnosis 

### 6.1 Diagnostic Algorithm

The most common presenting symptoms of TTS are acute chest pain, dyspnea, and syncope, which closely resemble those of ACS [8]. Meanwhile, patients presenting with ST-segment elevation should undergo urgent coronary angiography with left ventriculography to exclude an atherothrombotic event [8]. In contrast, clinical suspicion and pre-test probability assessment are key in non-ST-segment elevation presentations. An InterTAK diagnostic score of >70 points indicates a high likelihood. In such cases, coronary angiography is recommended for patients with atypical echocardiographic features, hemodynamic instability, or strong suspicion of ACS, whereas coronary computed tomography angiography (CCTA) may be considered in stable patients with typical TTS characteristics [8].

Currently, TTS is still approached as a diagnosis of exclusion, confirmed only after ruling out obstructive coronary disease. However, the diagnostic algorithm should shift toward a multimodal, physiology- and imaging-based assessment capable of identifying TTS features with greater sensitivity and specificity, even in the presence of concomitant coronary disorders (Fig. 2).

### 6.2 Diagnostic Tools 

Several clinical and instrumental features are highly suggestive of TTS (Table 3) [6].

**Table 3.** **Main diagnostic features of TTS**.

Diagnostic tool	Key features
12-lead ECG	Ischemic ST-segment and T-wave changes. ST-segment elevation in aVR combined with anteroseptal leads is highly specific for diagnosis. T-wave inversion and QT prolongation are specific for stress cardiomyopathy.
InterTAK score	Female sex
	+25 points
	Emotional trigger (e.g., severe emotional stress, bereavement, argument, emotional shock)
	+24 points
	Physical trigger (e.g., surgery, asthma attack, traumatic event, intense pain)
	+13 points
	Psychiatric disorder (documented in medical history, e.g., anxiety, depression, bipolar disorder)
	+11 points
	Neurological disorder (e.g., stroke, TIA, epileptic seizures, multiple sclerosis, etc.)
	+9 points
	Absence of ST-segment depression
	+12 points
	Prolonged QTc
	+6 points
	∙ <40 points: Low probability of TTS. The clinical presentation is more consistent with an acute coronary event or another cardiac condition.
	∙ 40–49 points: Borderline zone. Further evaluations are required to differentiate TTS from ACS.
	∙ ≥50 points: High probability of TTS. Correlate this score with clinical and instrumental findings to confirm the diagnosis.
	∙ 70 points: Very high probability of TTS.
Cardiac biomarkers	Troponin concentrations exceed normal in more than 90% of cases, although levels typically remain below 10 ng/mL. CK-MB demonstrates only a slight rise. Meanwhile, BNP/NT-proBNP levels are markedly elevated and can remain elevated for up to 3 months.
Echocardiography	This is the preferred noninvasive imaging modality, demonstrating widespread left ventricular akinesis or dyskinesis extending beyond a single coronary artery territory. Apical ballooning, often accompanied by LVOTO and mitral regurgitation arising from systolic anterior motion of the mitral valve, is frequently observed.
Coronary angiography	Typically performed on an urgent basis to exclude ACS, although it may not be required in all patients. Ventriculography is often performed to confirm stress cardiomyopathy, especially in midventricular presentations.
Cardiac MRI (CMR)	Enables direct assessment of myocardial edema (T1/T2 mapping) with an apical-to-base gradient. Absence of fibrosis through LGE imaging surpasses echocardiography in identifying left ventricular thrombi.
Coronary CTA	Employed for individuals with suboptimal echocardiographic windows or contraindications to cardiac magnetic resonance. This modality also facilitates evaluation of the epicardial coronary arteries, helping exclude significant stenotic lesions.

CMR, cardiac magnetic resonance; CK-MB, creatine kinase myocardial band; LV, left ventricle; LGE, late gadolinium enhancement; LVOTO, left ventricular outflow tract obstruction; MR, mitral regurgitation; TIA, transient ischemic attack; CTA, computed tomography angiography.

ECG abnormalities are observed in over 95% of cases, typically diffuse T-wave inversion and QTc prolongation, which appear 24–48 h after symptom onset. ST-segment elevation occurs in about 40% of patients and warrants urgent coronary angiography. A QTc prolongation can exceed 500 ms and increases the risk of ventricular arrhythmias. An ECG can also help differentiate TTS from ACS [33, 34]. ST-segment elevation in aVR, accompanied by ST-elevation in anteroseptal leads, has shown 100% specificity for TTS [35]. Low QRS voltage and amplitude attenuation are also frequent [36].

Cardiac troponins T or I are elevated in nearly all patients. The mismatch between mild biomarker elevation and extensive wall motion abnormalities reflects non-necrotic mechanisms of troponin release [37]. A recent study revealed that cTnT fragments (18 kDa) are the dominant form released in TTS. The ratio of long to total cTnT (troponin ratio) was lower in TTS patients than in MI patients, with strong discrimination between TTS and Type 1 MI (area under the curve (AUC): 0.869). However, troponin ratio is only a preclinical tool without a standardized dosing regimen [38].

Serum cardiac natriuretic peptides (B-type natriuretic peptide (BNP) and N-terminal pro B-type natriuretic peptide (NT-proBNP)) are usually higher than in STEMI and correlate with LV dysfunction and poor outcomes [39]. Thus, the BNP/troponin ratio may further aid differential diagnosis [40, 41]. Furthermore, a pooled analysis revealed significantly lower copeptin levels in TTS than in acute myocardial infarction (AMI), suggesting greater hemodynamic and neurohumoral stress in TTS [42, 43].

Transthoracic echocardiography remains the first-line tool for conducting a diagnosis, revealing transient regional akinesis or dyskinesis that extends beyond a single coronary territory. Apical ballooning is the most common presentation and may be accompanied by LV outflow tract obstruction (LVOTO) [6, 25]. Global longitudinal strain (GLS) and derived inferior–apical ratio (IAR) and inferior–lateral–apical ratio (ILAR) aid differentiation from anterior STEMI [44]. Advanced indices, such as myocardial work (MW), are also impaired in the apical segments despite recovery of left ventricular ejection fraction (LVEF), and correlate with in-hospital outcomes [45]. Echocardiography also enables thrombus detection and hemodynamic monitoring (noninvasive CO assessment from LVOT–VTI measurements).

Urgent coronary angiography is performed primarily to rule out ACS. However, in patients with typical TTS presentation and an unfavorable risk-benefit ratio due to comorbidities, noninvasive approaches may be prioritized [8]. Intravascular imaging (intravascular ultrasound (IVUS) and optical coherence tomography (OCT)) can identify plaque erosion, plaque rupture, and calcified nodules, helping TTS reclassifications as T1MI and spontaneous CAD.

**Fig. 3.**
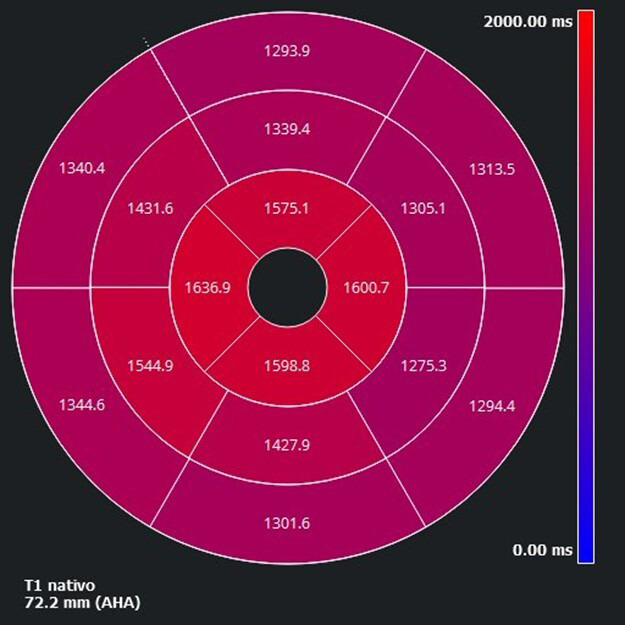
**Bullseye plot of the apical elevation of regional native T1 mapping values, consistent with myocardial edema in TTS**. The distribution illustrates typical apical involvement with a basal-to-apical gradient. AHA, American Heart Association.

CMR plays a central role in confirming TTS and excluding alternative causes of myocardial injury. T2-weighted imaging and T2 mapping sequences detect myocardial edema, often showing an apical-to-basal gradient [46, 47]. Extracellular volume (ECV) and native T1 values are also elevated in the affected regions, reflecting inflammation and an edema (Fig. 3) [48, 49]. In TTS, a myocardial edema usually appears diffuse and circumferential, unlike the territorial or subepicardial patterns seen in infarction or myocarditis [47]. The absence of late gadolinium enhancement (LGE) helps differentiate TTS from ACS and inflammatory cardiomyopathies, in which transmural or focal enhancement is common. LGE is typically faint and non-transmural when present in TTS [47, 50, 51]. Advanced strain analysis (two-dimensional (2D)/four-dimensional (4D) tissue-tracking CMR) presents the apical ballooning pattern and predicts recovery [46, 52, 53]. CMR is also sensitive for detecting LV thrombus [47]. Recent data from adenosine stress perfusion imaging have shown diffuse subendocardial perfusion defects and globally reduced myocardial blood flow (MBF), consistent with CMD [54].

CCTA and CCTA–fractional flow reserve (FFR) can be used in hemodynamically stable patients or in those with a high pre-test probability of TTS but an unfavorable invasive risk, allowing rapid exclusion of obstructive CAD or spontaneous coronary artery dissection (SCAD) [31, 55, 56].

Cardiac scintigraphy with ^123^I-β-methyl-iodophenyl pentadecanoic acid or cardiac positron emission tomography (PET) with ^18^F-FDG-PET demonstrates reduced perfusion and metabolism in dysfunctional regions [8, 57]. Quantitative PET imaging remains the gold standard for noninvasive assessment of CMD, enabling the segment-level evaluation of MBF flow reserve (MFR) and coronary vascular resistance (CVR) [58]. In a recent registry of 62 TTS patients, PET confirmed a high prevalence of TTS-related coronary microvascular dysfunction with partial recovery at follow-up despite normalization of LV function [59].

Recent data further challenge the diagnostic algorithm, showing that TTS and CAD can coexist, supporting a possible bidirectional effect. In addition, TTS shares multiple pathophysiological substrates with CMD syndromes—ANOCAs and INOCAs—highlighting the need for a precise differential diagnosis (Fig. 2).

## 7. Interplay Between TTS and ACS

The relationship between TTS and ACS is increasingly recognized as pathophysiologically interconnected rather than mutually exclusive. CAD frequently coexists with Takotsubo syndrome (Fig. 3) [60, 61]. Consequently, diagnostic criteria have been updated to allow a TTS diagnosis even in the presence of stable CAD, provided there is no acute plaque rupture or culprit lesion [2]. However, the hemodynamic and neurohormonal stress associated with ACS can precipitate TTS, creating overlapping phenotypes that complicate diagnosis [62].

In a large registry of 3506 ACS patients, 0.3% exhibited concurrent features of both ACS and TTS, including culprit plaque rupture and LV ballooning beyond the infarct-related territory (Table 4) [63]. Similarly, Chao *et al*. [64] reported that 26% of patients with acute left anterior descending (LAD) artery occlusion showed contraction patterns consistent with TTS. Meanwhile, TTS caused by ACS identified patients with wall abnormalities that did not correlate with the culprit coronary vessel. This entity is likely very underdiagnosed [65, 66]. Mechanistically, coronary vasospasm, transient ischemia, catecholamine surges, endothelial dysfunction, platelet activation, and local inflammation act synergistically, with physical and emotional stress amplifying sympathetic drive and myocardial stunning [62, 63]. Post-ischemic myocardial stunning (PIMS) and peri-infarct hyperkinesis can mimic the wall motion patterns of TTS [63]. In the series by Y-Hassan [65] 20 patients with PIMS exhibited ECG and imaging findings indistinguishable from TTS. OCT demonstrated thin-cap fibroatheromas in over 25% of cases, supporting the hypothesis of transient plaque rupture with spontaneous self-lysis as a potential mechanism [62]. ACS-related TTS predominantly involves the LAD artery, although an obtuse marginal or right coronary artery lesion can lead to midventricular variants [64]. CMR can reveal a segmental perfusion defect and transmural LGE complicating the differential diagnosis [67, 68].

**Table 4.** **Data derived from [63]**.

Total TTS patients (without plaque rupture/occlusion)	137
Total ACS patients	3506
Patients with coexisting ACS and TTS	9 (0.3%)
Coronary arteries involved	Right coronary artery (n = 3), circumflex artery (n = 3), mid-LAD (n = 2), ramus intermedius (n = 1)
PCI performed	A total of 7 patients (78%) of 9 patients with coexisting ACS and TS
Initial ejection fraction	26 ± 7%
Ejection fraction after resolution	57 ± 3%

ACS, acute coronary syndrome; PCI, percutaneous coronary intervention; TTS, Takotsubo Syndrome; LAD, left anterior descending artery.

Conversely, TTS may rarely precipitate ACS [51, 54]. Potential mechanisms include microvascular dysfunction, vasospasm, or SCAD caused by mechanical shear stress between hypercontractile and akinetic segments. Catecholamine-mediated thrombotic effects or embolization from apical LV thrombi may also lead to coronary occlusion [51].

## 8. TTS in the Minoca Spectrum

MINOCAs represents a working diagnosis encompassing patients who meet the clinical and biochemical criteria for AMI (ischemic symptoms and troponin elevation above the 99th percentile) without evidence of obstructive CAD (<50% stenosis). This heterogeneous group, representing 5–14% of all AMI cases, encompasses coronary causes (coronary spasm, microvascular dysfunction, plaque erosion, myocardial bridging, SCAD), non-coronary cardiac mechanisms (cardiac trauma, myocarditis, TTS, cardiomyopathies), and systemic conditions (sepsis, pulmonary embolism, stroke) [69, 70, 71].

Within this spectrum, TTS constitutes a distinct yet overlapping phenotype, frequently misclassified with other MINOCAs [72]. Early identification of TTS remains crucial for proper risk stratification and targeted therapy, as phenotypic overlap further complicates diagnosis.

**Fig. 4.**
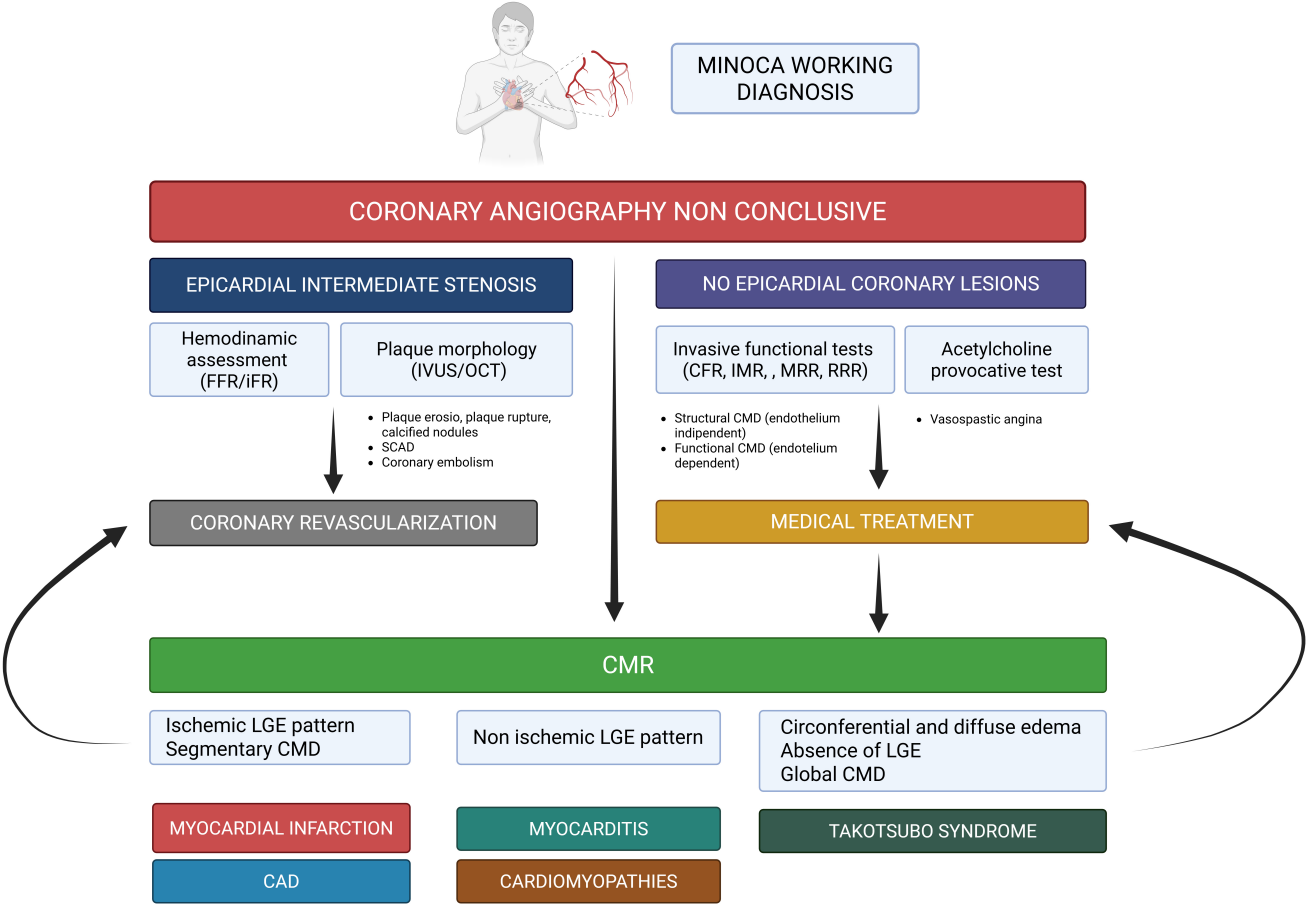
**TTS in the myocardial infarction with non-obstructive coronary artery (MINOCA) syndrome spectrum: a stepwise diagnostic approach**. FFR, fractional flow reserve; iFR, instantaneous wave-free ratio; IVUS, intravascular ultrasound; OCT, optical coherence tomography; LGE, late gadolinium enhancement; CFR, coronary flow reserve; IMR, index of microvascular resistance; MRR, microvascular resistance reserve; RRR, resistive reserve ratio; SCAD, spontaneous coronary artery dissection; CMD, coronary microvascular dysfunction; CAD, coronary artery disease. Figure created with BioRender.com.

When invasive angiography reveals no culprit lesion, a stepwise diagnostic approach is essential (Fig. 4) [71, 72]. Functional coronary assessment using FFR or instantaneous-wave free ratio (iFR) helps rule out hemodynamically significant stenoses. Intracoronary imaging (IVUS or OCT) can identify plaque rupture, erosion, or SCAD. Coronary functional testing, including coronary flow reserve (CFR), index of microvascular resistance (IMR), and acetylcholine provocation testing, explores microvascular dysfunction and endothelial function. Finally, CMR remains the cornerstone of MINOCA work-ups, enabling tissue characterization through T1/T2 mapping and LGE, thereby excluding alternative etiologies [70, 72].

Therefore, TTS should be recognized as a specific etiology within the MINOCA spectrum, not merely a diagnosis of exclusion. A multimodality, physiology-based diagnostic algorithm is essential to differentiate stress-induced myocardial dysfunction from true ischemic injury.

### TTS and SCAD

SCAD and TTS are major causes of non-atherosclerotic acute cardiac syndromes, both predominantly affecting women [73]. Importantly, differentiation is critical, as mechanisms, treatment, and prognosis differ substantially. Although both may follow adrenergic or emotional stress, TTS results from catecholamine-mediated myocardial stunning for direct myocyte toxicity and microvascular dysfunction. In contrast, SCAD arises from shear stress, including induced vascular injury, rupture of the vasa vasorum, and intramural hematoma [74]. Despite distinct mechanisms, clinical overlap is frequent, with both conditions presenting as stress-related chest pain, ECG alterations, and mild-to-moderate troponin elevations that mimic ACS. Furthermore, SCAD, involving the LAD, can produce transient apical dysfunction resembling TTS [75]. In the NYU Takotsubo Registry, up to 11% of patients initially diagnosed with TTS were reclassified as SCAD, and nearly one-third showed wall motion abnormalities extending beyond the dissected segment [76]. Phenotypically, TTS affects postmenopausal women with cardiovascular risk factors or anxiety disorders, whereas SCAD typically occurs in younger women, often associated with migraine, pregnancy, and fibromuscular dysplasia (FMD) [73, 77]. Thus, both conditions rarely coexist. Ischemic injury from coronary artery dissection may trigger myocardial stunning, such as PIMS, or the hyperkinetic basal segments in TTS may induce dissection in proximal coronary vessels [78, 79, 80].

Accurate differentiation requires multimodality imaging. Intracoronary imaging (IVUS/OCT) is essential to confirm SCAD, especially types 2–3 dissections, which may be angiographically silent. CMR identifies territorial edema with ischemic LGE patterns in SCAD, which contrasts with the circumferential edema without LGE in TTS.

Prognostically, SCAD generally carries a favorable long-term outcome but warrants vigilance for recurrence and extra coronary arteriopathies, justifying vascular screening and secondary prevention [81]. Conversely, TTS has higher in-hospital and long-term mortality, driven by systemic complications [73]. Hence, tailored secondary prevention, including β-blockers, antiplatelet therapy, and management of cardiovascular risk factors, is essential in SCAD, while TTS management focuses on acute complication prevention and recurrence reduction [73, 77].

## 9. TTS Associated With Chronic CAD 

### 9.1 Stable CAD

CAD was present in approximately 15% of cases in the International Takotsubo Registry, while larger registries report a prevalence of up to 60% [82, 83]. In a recent study of 1016 TTS patients, 23% had obstructive CAD, 41% non-obstructive CAD, and 36% normal coronary arteries on invasive angiography. In addition, 29% had luminal stenosis severity greater than 50% in at least one epicardial vessel [83]. The presence of obstructive CAD was associated with increased rates of cardiogenic shock, need for mechanical ventilation, and higher short-term mortality [84].

A pre-existing stable coronary lesion (chronic coronary syndrome, CCS) may limit CFR, reducing myocardial perfusion during stress, amplifying ischemia, and precipitating transient dysfunction beyond a single vascular territory. Thus, CAD acts as a pathophysiological amplifier of TTS.

**Fig. 5.**
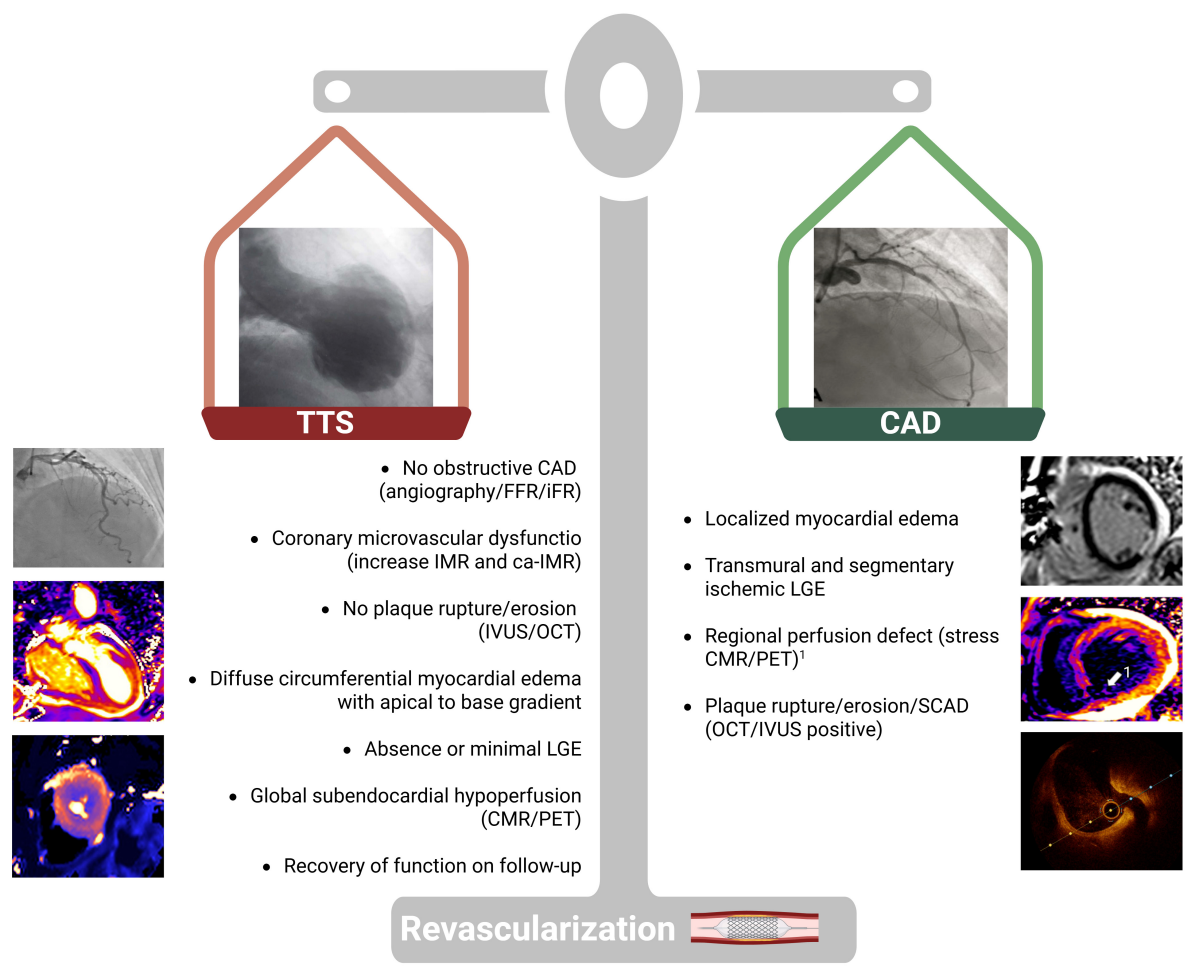
**Integrate physiology- and imaging-based diagnostic algorithm in patients with concomitant CAD**. FFR, fractional flow reserve; iFR, instantaneous wave-free ratio; IVUS, intravascular ultrasound; OCT, optical coherence tomography; LGE, late gadolinium enhancement; CMR, cardiovascular magnetic resonance; PET, positron emission tomography. Adapted from published studies [85, 86, 87, 88]. Figure created with BioRender.com.

The critical diagnostic challenge lies in discerning whether CAD represents a coexisting condition or a causal mechanism (Fig. 5, Ref. [85, 86, 87, 88]).

A morphological and functional coronary assessment is mandatory to determine the extent of coronary atherosclerosis (focal vs. diffuse), evaluate the functional significance of intermediate lesions (FFR/iFR), and assess plaque stability (IVUS/OCT). The exclusion of acute atherothrombotic events supports the diagnosis of TTS, even in the presence of stable CAD.

Management of coexisting CAD in TTS remains uncertain. In this setting, CMR is pivotal for differentiation. A localized edema or transmural ischemic LGE pattern favors the coexistence of significant CAD, whereas diffuse, circumferential edema without LGE supports TTS. Perfusion imaging (stress-CMR or PET) may further refine the distinction, revealing regional perfusion defects in CAD versus global subendocardial hypoperfusion typical of TTS. Meanwhile, myocardial revascularization should be considered in these cases [68].

In summary, the coexistence of CAD should not exclude TTS; however, the functional and prognostic impact of CAD must be carefully evaluated through integrated anatomic, physiologic, and imaging assessment to guide individualized management.

### 9.2 TTS and Coronary Microvascular Dysfunction

A growing body of evidence supports the central role of CMD in the pathophysiology of TTS [31, 89]. CMD represents a shared pathophysiological substrate linking TTS with ANOCAs and INOCAs, accounting for the frequent clinical overlap among these disorders (Table 5). Patients in these groups commonly present with stress-related chest pain, non-obstructive coronaries, and objective signs of ischemia, particularly in INOCAs, which can mimic early or recurrent TTS [54, 90, 91]. However, TTS is distinguished by acute onset, transient wall motion abnormalities extending beyond a single coronary territory, and reversible microvascular impairment.

**Table 5.** **TTS and coronary microvascular dysfunctions (CMDs): a comparative table between TTS, ischemia with non-obstructive coronary disease (INOCA), and angina with non-obstructive coronary disease (ANOCA)**.

Clinical feature	TTS	INOCAs	ANOCAs
Definition	Acute and reversible form of left ventricular dysfunction in the absence of significant obstructive CAD during acute stress.	Evidence of myocardial ischemia in the absence of obstructive CAD.	Anginal symptoms or ischemic equivalent despite non-obstructive CAD and absence of noticeable ischemia.
Pathophysiological hallmark	Catecholamine-induced myocardial injury.	Chronic ischemia due to impaired coronary microvascular or vasomotor function.	Chronic angina due to impaired coronary microvascular or vasomotor dysfunction.
Type of CMD involved	Functional CMD (endothelium-independent) in early TTS.	Functional CMD (endothelium-independent) from an inappropriate arteriolar vasodilatory response.	Predominantly functional CMD, with structural dysfunction in selected cases.
	Structural CMD (endothelium-dependent) in late TTS.	Structural CMD (endothelium-dependent) from increased microvascular resistance.	
Typical trigger and symptomatology	Intense emotional or physical stressor-induced angina, dyspnea.	Exercise-induced or emotional stress-related myocardial ischemia.	Emotional stress or strain causes angina, dyspnea, weakness, nausea, and/or irregular sleep patterns.
Troponin elevation	Elevated (>99th percentile).	Mildly elevated or normal depending on ischemic burden.	Normal or minimally elevated.
ECG	ST-deviations, T-wave inversion, QTc prolongation.	Transient ST-depression and T-wave inversion.	Normal or minimal aspecific changes.
Coronary angiography	Non-obstructive CAD (<50% stenosis), no culprit lesion.	Non-obstructive CAD, microvascular, or vasospastic etiology suspected.	Non-obstructive CAD, epicardial spasm, or microvascular dysfunction may be present.
Echocardiography	Diffuse regional LV dysfunction beyond a single coronary territory (e.g., apical ballooning).	Rarely found.	Rarely found.
Cardiac MRI (CMR)	Diffuse and circumferential myocardial edema without LGE; global microvascular dysfunction.	Mild perfusion defects. Rarely mid-wall or patchy LGE. Segmental microvascular dysfunction.	Normal findings, without LGE.
Invasive coronary physiology:			
coronary flow reserve (CFR);	CFR: Reduced (often <2.0), especially in the acute phase.	CFR: Reduced (often <2.0), reflecting impaired vasodilatory capacity.	CFR: Mildly to moderately reduced.
microvascular resistance (IMR);	IMR: Elevated in the acute episodes with regression in the recovery phase.	IMR: Elevated (often >25) in structural CMD; normal (<25) in functional CMD.	IMR: Elevated in underlying structural CMD.
microvascular resistance reserve (MRR)	MRR: reduced (<2.7) in the acute phase.	MRR: reduced (<2.7) in functional CMD.	
Clinical course	Self-limited and reversible.	Chronic and relapsing.	Chronic, with persistent or intermittent symptoms.
Treatment	Supportive: β-blockers, stress reduction, avoid catecholamines, diuretics, and anticoagulation in selected cases.	Lifestyle changes, control CV risk factors, b-blockers, CCB, Ranolazine, trimetazidine, Ivabradine, nitrates, Nicorandil.	Lifestyle changes, control CV risk factors, b-blockers, CCB, Ranolazine, trimetazidine, Ivabradine, nitrates, Nicorandil.

CAD, coronary artery disease; CMD, coronary microvascular dysfunction; TTS, Takotsubo syndrome; LV, left ventricle; LGE, late gadolinium enhancement; CMR, cardiovascular magnetic resonance; CFR, coronary flow reserve; IMR, microvascular resistance; MRR, microvascular resistance reserve; CCB, calcium channel blocker.

Because TTS may present with subtle or atypical features, particularly in recurrent or subacute forms, the differential diagnosis can be challenging. Thus, a comprehensive multimodal assessment remains essential for accurate differentiation and tailored management.

In ANOCAs/INOCAs, functional testing identifies endotypes involving either endothelium-dependent or endothelium-independent mechanisms, each with prognostic and therapeutic relevance [31]. Functional CMD, often seen in microvascular angina or early TTS, is characterized by elevated resting coronary flow and impaired hyperemic response, reflecting smooth muscle or autonomic dysregulation. In contrast, structural CMD, as seen in cardiomyopathies, HF, or late TTS, involves endothelial dysfunction and vascular remodeling, leading to increased microvascular resistance and reduced CFR [71].

Noninvasive imaging provides key diagnostic insights. Stress echocardiography, stress PET, perfusion CCTA–CFR, and CMR can quantify microvascular function through CMR-derived myocardial perfusion reserve index (MPRI) or stress MBF [31, 71, 90, 91]. In a CMR study of 42 patients with TTS, more than 80% exhibited microvascular dysfunction during the acute phase, which correlated with LVEF and myocardial edema [92]. Cardiac PET confirmed diffuse subendocardial hypoperfusion and reduced MBF under stress, with partial recovery at follow-up [59].

Invasive coronary physiology remains the gold standard, while CFR represents the most commonly used parameter [90]. CRF is defined as the ratio of CBF during hyperemia against the value at rest and describes the ability of the coronary tree to increase MBF in response to increased oxygen demand. These tests can also be performed during the index coronary angiography procedure with accurate and safe results [71]. CMD is defined by a CFR <2.0, microvascular resistance (IMR) >25, obtained with thermodilution, or myocardial resistance reserve (MRR) >2.7, through the quantitative angiography method [31, 71]. An early invasive study found elevated IMR values in both TTS and STEMI patients, suggesting comparable microvascular dysfunction, albeit transient in TTS, and the absence of irreversible necrosis [93]. In a prospective study, IMR values inversely correlated with the time from symptom onset, confirming progressive microvascular recovery [94]. Likewise, elevated angiography-derived IMR (caIMR), particularly in those with LAD, has been documented during the acute phase and found to normalize during follow-up [57].

Aggregated evidence indicates that CMD is far more prevalent in patients with TTS (~80%) than in those with INOCAs (~20%), with higher IMR, lower CFR, and reduced resistive reserve ratio (RRR) values, particularly in the apical variant [89, 95, 96, 97]. In the largest pooled analysis (166 patients, nine cohorts), elevated IMR values during the acute phase independently predicted all-cause mortality and MACCEs at the 20-month follow-up [98].

Randomized controlled trials (RCTs) directly comparing TTS with CMD-related disorders remain limited. Moreover, further prospective studies are warranted to elucidate the therapeutic potential of CMD and to refine individualized management strategies.

## 10. Cardiac Complications and TTS Outcomes

Although transient in nature, TTS carries a substantial risk of acute complications, occurring in approximately 20% of patients, with an in-hospital mortality of about 4% [2]. Despite apparent LV function recovery, long-term outcomes mirror those of ACS, with recurrence in up to 20% over 10 years and comparable mortality during follow-up [2, 99, 100].

Thus, accurate and timely diagnosis is pivotal, as it directly influences the management of acute complications, particularly arrhythmias, LVOTO, and thromboembolic events, which require dedicated therapeutic strategies distinct from those used in ACS.

Arrhythmic events occur in 4–10% of TTS cases, most commonly ventricular tachycardia (VT) or ventricular fibrillation (VF), and serious arrhythmias, including VT/VF, high-degree atrioventricular (AV) block, and sick sinus syndrome, have been reported in 6.2% of 16,000 registry patients [2, 101, 102]. Hypotension is the leading cause of sudden cardiac death (SCD) during the acute phase, whereas between 24 and 72 hours, prolonged QTc may precipitate torsades de pointes [103, 104]. The arrhythmogenic substrate reflects catecholamine-induced calcium overload, microvascular dysfunction, and myocardial edema, which promote delayed afterdepolarizations, re-entry circuits, and triggered activity. Additionally, catecholamine-driven abnormal automaticity and conduction disturbances, such as complete AV block and sinoatrial block, have been described [105]. A recent study found that a QTc >490 ms, apical ballooning, and severe LV dysfunction are strong predictors of VT/VF, particularly within the first 24 hours [106]. Del Buono *et al*. [107] reported that QTc >460 ms increases arrhythmic risk by 21%, while each increase of 10 ms raises arrhythmic risk by 9%. Prior stroke or transient ischemic attack (TIA) and vasopressor therapy represent additional risk factors [107]. A global Tpeak–Tend interval of >108 ms can improve predictions of subacute ventricular events [108]. Hemodynamic ventricular arrhythmias require DC shock; drugs include BBs, amiodarone, and lidocaine in refractory cases. Polymorphic VT can be treated with magnesium sulphate, temporary pacing, and lidocaine [105]. Meanwhile, implantable defibrillators may be considered for life-threatening ventricular arrhythmias, although long-term data in TTS are limited. Wearable defibrillators offer a temporary alternative for arrhythmia protection and monitoring during recovery in those with residual arrhythmic state or concomitant conditions may be playing a role [8, 105, 109]. Finally, the one-year mortality was significantly higher in patients experiencing life-threatening arrhythmias compared with patients without arrhythmias [101].

Atrial fibrillation (AF) occurs in 5–15% of TTS patients, usually in a paroxysmal form, driven by elevated left atrial pressure, inflammation, and catecholamine [2, 110]. Impaired left atrial strain is also associated with AF occurrence and in-hospital complications [111]. AF burden also correlates with worse outcomes both during hospitalization and long-term follow-up [112, 113]. Management requires cautious rate and rhythm control with beta-blockers and/or digoxin, avoiding amiodarone and sotalol if the QTc is prolonged [105].

In cases of high-degree AV block with hemodynamic compromise, temporary pacing may be necessary, but permanent PMK implantation should be deferred until LV function recovers. Isoproterenol should be used with caution [105, 109].

Acute HF is the most common complication, occurring in 12–45% of patients [2]. Echocardiography reveals reduced LVEF without significant LV dilation. In approximately 13% of cases, acute HF is exacerbated by dynamic LVOTO, which increases intraventricular gradients, decreases stroke volume, and may cause secondary mitral regurgitation [2]. In a recent multicenter study of 322 patients with TTS complicated by cardiogenic shock, LVOTO was identified in 18% of cases, associated with greater in-hospital morbidity, including ventricular arrhythmias, bleeding, and acute kidney injury, but similar 90-day and 5-year mortality rates [114].

**Fig. 6.**
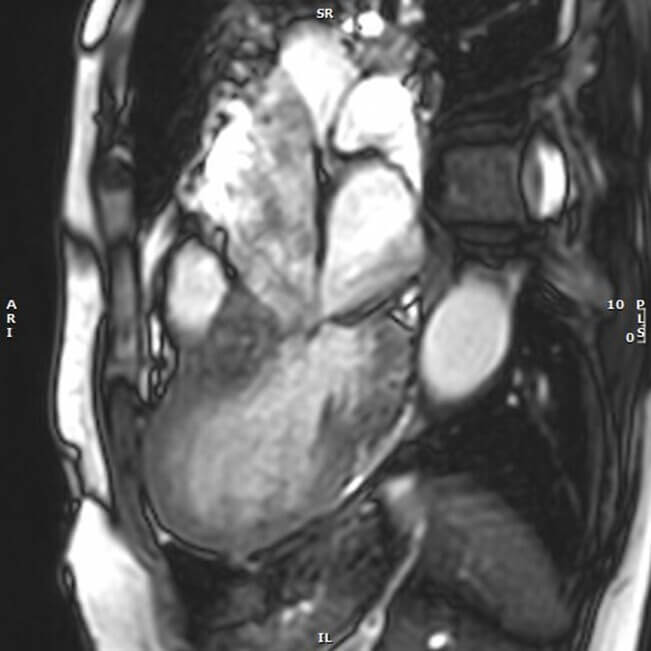
**CMR imaging: cine bSSFP end-systolic frame of TTS-related systolic anterior motion of the anterior leaflet of the mitral valve resulting in Carpentier type IV secondary mitral regurgitation**. bSSFP, balanced Steady-State Free Precession.

Acute secondary mitral regurgitation results from systolic anterior motion (SAM) of the mitral valve or papillary muscle tethering, further impairing LV systolic performance. These mechanisms are well characterized by CMR cine imaging (Fig. 6).

Cardiogenic shock, although less frequent (10–15%), represents the most severe manifestation driven by extensive LV dysfunction, dynamic LVOTO, right ventricular involvement, or severe mitral regurgitation. Catecholamine-based agents may worsen LVOTO, increase myocardial oxygen demand, and exacerbate myocardial injury [114]. In the RETAKO registry, mortality reached 13% at 90 days and 22% at 5 years, with prognosis determined primarily by shock severity rather than sex [115]. Comprehensive hemodynamic assessment—ideally integrating echocardiography and invasive monitoring—along with phenotyping based on clinical severity, LVEF, mechanical ventilation, infection status, and hospital course, remains essential to guide individualized management [114].

**Fig. 7.**
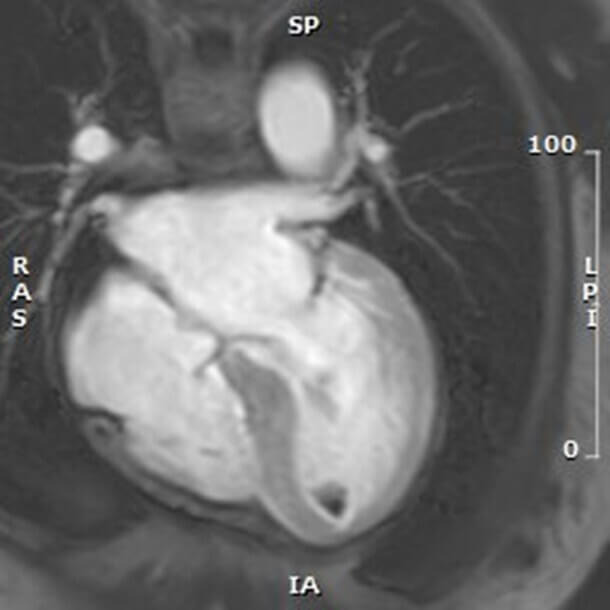
**Early post-contrast T1-weighted CMR image in a 4-chamber view of the apical hypointense lesions consistent with intraventricular thrombus, as a complication of TTS**.

LV thrombus is a recognized complication of TTS, particularly in apical variants, with an incidence of 1.3% to 9.3% [116]. In the GEIST Registry, 2.2% of patients with apical ballooning developed LV thrombi, which were associated with higher ST-elevation and peak TnI levels. The highest risk period is known to occur 2–5 days after symptom onset, when LV systolic dysfunction is at its peak. Cerebrovascular embolic events occur in up to 17% of these patients [117]. Thrombus formation results from blood stasis in akinetic segments, catecholamine-mediated endothelial injury, and a hypercoagulable state. Serial echocardiographic evaluations and cardiac MRI (CMR) are recommended for surveillance in high-risk patients. CMR remains the gold standard for thrombus detection, revealing avascular masses without contrast enhancement (Fig. 7). Advanced CMR techniques improve sensitivity for small or mural thrombi, which are often missed by standard imaging. Protruding or mobile thrombi carry the highest embolic potential. Data from multicenter registries confirm that ischemic stroke occurs in approximately 2–4% of patients during the index hospitalization, with risk persisting in the subacute phase. These findings underscore the importance of vigilant imaging follow-up and individualized anticoagulation strategies.

## 11. Treatment

The management of TTS remains largely consensus- and registry-based, as RCTs are lacking, contributing to heterogeneous treatment strategies. Therapy focuses on eliminating trigger stressors, preventing arrhythmic and hemodynamic complications, and supporting ventricular recovery. Drugs that prolong QTc or induce bradycardia should be discontinued to lower the risk of VAs. Electrolyte imbalances, avoidance of catecholamine, and treatment of concurrent infections are also fundamental components of supportive care [6, 30].

Beta-blockers are recommended to mitigate sympathetic overactivation, with potential benefits in reducing arrhythmic risk and death, particularly in patients with hypertension and in those who have developed cardiogenic shock [118]. However, routine chronic use remains debated due to a lack of RCTs. Data from The GEIST Registry demonstrated, after propensity score matching, that beta-blockers were associated with a significant reduction in all-cause mortality, particularly within the first year. However, these benefits did not translate into reduced recurrence or faster LV recovery [119]. The most recent meta-analysis of 19 studies (n = 11,167) confirmed a 28% reduction in all-cause mortality and a 29% reduction in recurrence after 2 years, with a time-dependent benefit [120]. The ongoing β-Tako RCT will provide the first prospective data on the efficacy and safety of beta-blockers in TTS, using an echocardiographic wall motion score index at 7 days as the primary endpoint and LVEF, strain recovery, and 1-year MACEs as secondary outcomes [121].

Although the International Takotsubo Registry reported improved survival with RAAS inhibitors, more recent evidence from a propensity score analysis of the GEIST registry suggests that the benefit is restricted to patients with a reduced LVEF (≤40%) or diabetes [122, 123]. Furthermore, a recent systematic network meta-analysis found no synergistic effect of combined beta-blockers and ACEi/ARBs on recurrence [124]. In a case–control study, cardiovascular therapies have shown inconsistent survival benefits in TTS patients compared to patients with AMI, with worse outcomes reported using diuretics, anti-inflammatory, and psychotropic therapies [125].

Antithrombotic therapy is often initiated empirically due to the clinical overlap with ACS. However, meta-analyses and registries show no long-term benefit of single- or dual-antiplatelet treatment [126, 127]. The most recent meta-analysis demonstrated that long-term antiplatelet therapy may even increase the occurrence of MACEs and mortality without lowering recurrence, stroke/TIA, or MI rates [126]. Routine long-term antiplatelet therapy is not recommended unless there is concomitant CAD or another clear indication.

For acute HF, loop diuretics and oxygen may be used to alleviate congestion, but nitrates are contraindicated in LVOTO due to the risk of worsening obstruction. In CS without LVOTO, non-catecholaminergic inotropes (*i*.*e*., dobutamine, milrinone, or levosimendan) may be considered to improve cardiac output [128, 129, 130, 131]. Among these, levosimendan, a calcium sensitizer, enhances contractility while reducing afterload, with observational evidence of faster LV recovery and shorter hospitalization [132, 133]. If hypotension occurs, a combined approach with vasopressors, such as low-dose norepinephrine and, especially, vasopressin, may be cautiously added. In LVOTO-related shock, inotropes are contraindicated because they exacerbate obstruction by increasing basal hypercontractility. Management includes IV fluid, short-acting beta-blockers (metoprolol, esmolol, landiolol), and pure vasoconstrictors (phenylephrine, vasopressin, angiotensin II) that increase afterload without stimulating β-receptors [128]. The vasoactive–inotropic score (VIS) is an emerging prognostic tool: in the RETAKO analysis, mortality increased dramatically with rising VIS from 4% to 47%. Elevated VIS also predicted 30-day and 1-year mortality rates, acute kidney injury, and major bleeding, underscoring the need for early risk stratification and cautious vasopressor titration [134]. Mechanical circulatory support, such as a percutaneous ventricular assistance device (Impella) or VA-ECMO, may be considered in refractory cardiogenic shock to reduce LVEDP and LVOTO, increase CPO, and improve LV recovery, based on multicenter experience [135, 136]. In the RETAKO Registry, MCS was required in 9.6% of TTS patients with cardiogenic shock, and, despite higher complication rates, adjusted mortality was not increased, suggesting a potential benefit in selected cases [137]. Conversely, intra-aortic balloon pump (IABP) did not improve survival in the GEIST data [131]. Therefore, future prospective studies should clarify optimal timing, device selection, and patient profiles.

In TTS with concomitant stable non-obstructive and non-culprit CAD, aspirin and lipid-lowering therapy should be considered; however, no registry or randomized trials have demonstrated a reduction in TTS recurrence or major adverse cardiovascular events (MACEs) through coronary revascularization. For CAD-induced TTS, complete revascularization and guideline-directed medical therapy should be pursued when ischemia or plaque erosion is demonstrated [70].

Currently, no pharmacological strategy has proven effective in improving CMD or a long-term outcome in patients with TTS with concomitant CMD. Ongoing translational research aims to address CMD as a core pathophysiologic target, representing a major unmet therapeutic need.

For thromboembolic prevention, the European position paper recommends oral anticoagulation (OAC) for at least 3 months in patients with LV thrombus and considers OAC in high-risk TTS patients (age ≥75 years, EF <35%, LVOTO, or moderate to severe mitral regurgitation) until LVEF recovers. Therapy may be discontinued after three months if imaging confirms thrombus resolution [8, 117]. Meanwhile, OAC initiation between 48 hours and 15 days after the event is reasonable in patients with TTS-associated ischemic stroke (as part of the stroke–heart syndrome spectrum), depending on hemorrhagic risk [8, 117].

## 12. Conclusions and Future Directions

TTS is a distinctive yet underrecognized entity within the spectrum of ACSs, particularly among patients without MINOCA disease. In addition to its transient course, TTS is a complex cardiovascular condition driven by dynamic interactions between neurohormonal activation, CMD, and—in many cases—CAD. This interplay challenges the conventional paradigm of TTS as a mere “diagnosis of exclusion” and supports its reinterpretation as a distinct pathophysiological process with variable ischemic substrates.

The coexistence of TTS with CAD, SCAD, or CMD underscores the limitations of current diagnostic criteria. The presence of CAD or LGE should not preclude the diagnosis of TTS, as these entities often overlap or precipitate one another.

Future diagnostic frameworks should integrate an advanced physiology- and imaging-based diagnostic algorithm with intracoronary imaging, coronary physiology tests, and myocardial tissue characterization to define the predominant mechanism, whether ischemic, stress-related, CAD, or microvascular dysfunction, to accurately position patients along the ischemic stress cardiomyopathy continuum and to guide revascularization decisions (Fig. 2).

TTS should no longer be regarded as a benign or self-limiting response to stress, but rather as a distinct trait. The accurate recognition of TTS has immediate therapeutic relevance, enabling the prevention and management of potentially fatal complications such as arrhythmias, cardiogenic shock, and thromboembolism, because these disorders need a dedicated, mechanism-specific treatment approach, distinct from ACS management.

Current treatments remain largely empirical, and robust evidence is urgently needed. RCTs should assess the efficacy of neurohormonal blockade, microvascular-targeted therapies, and secondary prevention strategies tailored to individual pathophysiological profiles. The identification of TTS phenotypes with persistent CMD or concomitant CAD may pave the way for personalized management. Finally, large-scale multicenter registries combining morphological and functional intracoronary imaging are needed to validate the prognostic impact of coronary disease and microvascular dysfunction and to guide future targeted interventions.

## Abbreviations

ACS, Acute Coronary Syndrome; AF, Atrial Fibrillation; AMI, Acute Myocardial Infarction; ANOCA, Angina with Non-Obstructive Coronary Arteries; βAR, Beta-Adrenergic Receptor; BNP, B-type Natriuretic Peptide; CAD, Coronary Artery Disease; CMD, Coronary Microvascular Dysfunction; CK-MB, Creatine Kinase Myocardial Band; CMR, Cardiac Magnetic Resonance Imaging; ESC, European Society of Cardiology; ECG, Electrocardiogram; ET-1, Endothelin-1; FFR, Fractional Flow Reserve; iFR, Instantaneous Wave-Free Ratio; GLS, Global Longitudinal Strain; LBBB, Left Bundle Branch Block; LV, Left Ventricle; INOCA, Ischemia with Non-Obstructive Coronary Arteries; IL-6, Interleukin-6; IVUS, Intravascular Ultrasound; InterTAK, International Takotsubo Registry Diagnostic Criteria; LAD, Left Anterior Descending Artery; LVEF, Left Ventricular Ejection Fraction; LVOTO, Left Ventricular Outflow Tract Obstruction; MINOCA, Myocardial Infarction with Non-Obstructive Coronary Arteries; MIBG, Metaiodobenzylguanidine; MW, Myocardial Work; NT-proBNP, N-terminal pro B-type Natriuretic Peptide; OCT, Optical Coherence Tomography; PET, Positron Emission Tomography; QTc, Corrected QT Interval; SAM, Systolic Anterior Motion (of the mitral valve); SCAD, Spontaneous Coronary Artery Dissection; SPECT, Single-Photon Emission Computed Tomography; TTS, Takotsubo Syndrome; TdP, Torsades de Pointes; TIA, Transient Ischemic Attack; VWF, Von Willebrand Factor.
